# The sad plight of cell therapy for heart failure: causes and consequences

**DOI:** 10.20517/jca.2022.02

**Published:** 2022-03-02

**Authors:** Roberto Bolli, Xian-Liang Tang

**Affiliations:** Institute of Molecular Cardiology, University of Louisville, Louisville, KY 40292, USA.

**Keywords:** Cell therapy, heart failure, stem cells, progenitor cells, cardiac repair

As extensively reviewed in recent articles^[1,2]^, cell therapy has emerged as a promising new approach to the treatment of heart failure (HF). Unfortunately, the process of translating this therapy into a clinical tool has slowed dramatically in recent years, and now appears to be almost at a standstill^[3]^. Paradoxically, this has happened at a time when clinical trials of cell therapy in chronic HF have yielded the most encouraging results^[2]^. What went wrong? Can anything be done to overturn the impasse? In this essay, we will expose the current misconceptions regarding cell therapy in HF and highlight the fundamental problem - conflating the outcome of trials in HF with that of trials in ST-elevation myocardial infarction (STEMI). We will discuss why the present situation is both scientifically irrational and medically harmful to the health of countless patients who suffer from HF, and why things must change. We will argue that the current hostility toward this line of research will have negative consequences not only for our understanding of the potential utility of cell therapy, but also for the treatment of HF patients. But first, let’s survey the extent of the damage inflicted on a very promising field of research.

## CURRENT SITUATION

For investigators interested in the use of cell therapy for HF, these are the dark ages. Indeed, the current state of affairs is the bleakest it has ever been. At the clinical research level, no major funding organization is currently willing to support clinical trials of cell therapy for HF in adults. Even applications that receive good scores are not funded. To compound the matter, most biotechnology and pharmaceutical companies have followed suit and made a strategic decision to eschew investments in trials of cell therapy for HF. The dearth of public or private funding means that few clinical trials are currently ongoing in this field, and even fewer are planned. Things are not better at the basic research level, where it has become obvious to most investigators that projects focused on cell therapy for HF are unlikely to be funded, almost dead on arrival, so that even applying is futile. At the same time, as if in a well-orchestrated chorus, the editors of basically all major journals now meticulously avoid publishing papers that deal with cell therapy for heart disease in general, and HF in particular; such papers are flagged and, unless they cast a negative light on cell therapy, they are carefully eschewed, like the bubonic plague. The chorus of negativity, in turn, is picked up and amplified by the news media. All of this is a striking change from just a few years ago. How did we get to this point?

Specifically, is the decision not to invest in cell therapy for chronic HF justified by scientific evidence? The short answer is a resounding “no”. We have recently reviewed in detail the field of cell therapy in patients with chronic HF, highlighting the fact that several recent Phase II trials have yielded encouraging, even exciting results^[1,2]^. Based on this analysis, it is apparent that the ostracism by journals and the lack of funding for basic or clinical trials of cell therapy in HF cannot be explained by existing data and available evidence. It must, therefore, be dictated by other factors, discussed below.

Before starting our discussion, a few points are in order. In this essay, we will use the term “cell therapy” instead of “stem cell therapy” because, as we have pointed out before^[1]^, most of the cells used for the treatment of HF are not, or at least have not been proven to be, genuine stem cells in the strict sense of the term. Importantly, this article is concerned with chronic HF, not with acute or subacute HF resulting from a recent STEMI. Finally, for the purpose of the present discussion, the term “HF” refers to HF with reduced ejection fraction (HFrEF), because all major studies of cell therapy conducted heretofore have targeted HFrEF patients.

## WHY THERE IS OPPOSITION TO STUDIES OF CELL THERAPY FOR HF

Why is there widespread hostility toward cell therapy for HF? Its opponents (who are legions) have raised several criticisms, which are enumerated and discussed below:

### 1. Initial claims of regeneration have not been borne out.

Early studies claimed that cell therapy regenerates new cardiac myocytes, which replace dead or scarred tissue^[4,5]^. Later, however, several groups, including our own^[6-25]^, showed that this is not the case and that virtually all exogenous cells transplanted into the heart disappear rapidly, without forming new cardiac myocytes^[26]^. Specifically, we were the first group to show that c-kit-positive cardiac cells do not engraft and do not regenerate myocytes; over the past decade, we have published a large number of original papers^[6-8,12,13,15,17-25]^ and review articles^[8-12]^ making this point.

New insights and explanations are what move science forward and should be celebrated. Not so for cell therapy. Although in other fields, a change of understanding is usually viewed as progress, in the case of cell therapy, this change has been used as an argument to undermine the scientific validity and therapeutic promise of this approach. Such a position is contrary to the scientific method. Science is a self-correcting process in which ideas are continuously challenged and often found to be incorrect and replaced with new ideas. Thus, our new, improved understanding of the mechanism of action of cell therapy should be welcomed as an advance, not used to discredit cell therapy.

### 2. The mechanism of action is unknown.

Perhaps the most common objection to cell therapy for HF (and heart disease in general) is that the mechanism of action remains a mystery. This is true. All we can say at present is that transplanted cells improve cardiac function by releasing unknown factor(s) in the surrounding tissue or, perhaps, in the bloodstream, and that these factors *somehow* result in improved cardiac performance. Obviously, this is a very vague paradigm that falls short of explaining the beneficial actions of cell therapy. Even the currently fashionable (but as yet unproven) immune response hypothesis^[27]^ does not illuminate the exact mechanism whereby triggering an inflammatory response should improve cardiac performance.

It is our opinion that the mechanism of action of exogenous cells is multifactorial and extremely complex, and that it will be very difficult, if not impossible, to pinpoint one specific factor or signaling pathway that is uniquely responsible for the therapeutic actions of the cells. Considering that transplanted cells have been found to secrete myriad factors ranging from bioactive lipids to a panoply of non-coding RNAs, proteins, cytokines, chemokines, exosomes, and other extracellular vesicles, trying to ascribe the beneficial effects of cells to one factor may be futile^[12]^. Nature usually works through multiple and often redundant pathways. This is not to say that research on the mechanism of cell therapy should not continue. Our point is that clinical trials testing the efficacy and safety of cell therapy in HF should not wait until the mechanism is elucidated, which may take a very long time, or may even be impossible.

On the other hand, there are countless examples of therapies that are widely used in clinical medicine despite the fact that their mechanism of action is not understood. For example, statins are routinely prescribed for patients with acute coronary syndromes despite our ignorance of the mechanism(s) whereby they improve clinical outcomes in this setting. Nor is the mechanism of action of sodium-glucose transporter-2 inhibitors in HF known. Nevertheless, these therapies are very beneficial to patients. As is the case for other therapies, the requirements for clinical application of cell therapy should be the demonstration of safety and effectiveness, independently of whether we fully understand the molecular mechanism of action^[1,12]^.

### 3. Cell therapy has not worked in STEMI.

A third problem with cell therapy is the conflation of studies in chronic HF with studies in other settings. Even though the early clinical trials were not done in chronic HF, their outcome was extrapolated to chronic HF. From 2001 to a few years ago, the vast majority of studies of cell therapy for heart disease were conducted in patients with STEMI^[1]^. Unfortunately, all major trials conducted in this population over the past decade have been negative^[28]^, and the largest of them, the BAMI trial, was inconclusive^[29]^ (due to the dramatic improvement in the prognosis of STEMI patients in the past 30 years or so). This does not necessarily mean that cell therapy is not efficacious in STEMI, but rather, that the outcome of STEMI has improved to a point where demonstrating a benefit with cell therapy or, for that matter, with any therapy is extremely difficult if not impossible^[28]^.

Patients with STEMI are obviously very different from those with chronic HF. Unfortunately, as we have pointed out^[28]^, the failure to demonstrate a benefit of cell therapy in one specific clinical setting, STEMI, has been erroneously extrapolated to conclude that cell therapy is ineffective in *all* clinical settings of heart disease including chronic HF. Such conclusion is unwarranted and is refuted by the available evidence^[1]^. In fact, a careful review of the literature supports the notion that clinical research on cell therapy for HF is promising and should continue^[1,2]^.

### 4. Clinical trials have been misrepresented as showing “disappointing”, “minor”, or “incremental” results.

If you read just about any review, original paper, or editorial on cell therapy, you will find eerily similar statements to the effect that clinical trials of cell therapy have been “disappointing” or have yielded “minor” or “incremental” results. This assertion is assumed to be so obvious that it is stated as a premise, as a fact, not as a thesis to be demonstrated. It’s a given. Naturally, after reading this statement over and over, most people will come to believe that it must be true, because everyone says it.

The widespread acceptance of this misrepresentation is an excellent example of the proclivity of human beings to adopt a herd behavior. After all, following the herd is much safer and easier than thinking critically for oneself. And if the high-profile author of that editorial or review in a prominent journal says so, it must be true, right? Surely that author, being an “opinion leader”, knows what he/she is talking about. Besides, arguing with a powerful person could be costly, in many different ways. And so, the mantra that clinical trials of cell therapy for HF have been “disappointing” continues to be repeated in the literature.

There is only one problem - it’s not true. In fact, as pointed out in a recent review^[1]^, several Phase II/III, double-blind, randomized, multicenter clinical trials have revealed substantial beneficial effects of cell therapy in HF. In the setting of ischemic HF, these include ixCELL-DCM^[30]^, MSC-HF^[31]^, CONCERT-HF^[32]^, DREAM-HF^[33,34]^, and TAC-HFT^[35]^. The benefits of cell therapy range from a 6.2 units increase in left ventricular ejection fraction in MSC-HF^[31]^ (which is more than the average increase after thrombolytic therapy in STEMI or after CRT in HF) to an improvement in quality of life (measured by the MLHFQ score) and 6-min walk distance (TAC-HFT^[35]^) and to a reduction in major adverse cardiac events (MACE) by 37% (ixCELL-DCM^[30]^), 77% (CONCERT-HF^[32]^), and 33%-65% (depending on the type of MACE) in DREAM-HF^[33,34]^. Certainly, these trials are not “disappointing”, nor are the aforementioned salubrious effects “minor” or “incremental”. In fact, considering that the treatment was given *only once* and the outcome measured 12 months later, these effects are quite remarkable. Those who deny it should name another therapy that produces any measurable change in clinical status in HF patients 12 months after one dose. It is time for this widespread misrepresentation to be rectified.

### 5. Cell therapy has been tainted by scientific misconduct.

Finally, a major (albeit often unspoken) reason for the current dearth of cell therapy studies in HF is no doubt the discovery, a few years ago, of scientific misconduct in one prominent basic research laboratory^[36]^. The occurrence of data manipulation is always a tragedy in science. Nevertheless, it should be recognized that this tragic incident does not undermine the credibility of myriads of principled basic, translational, and clinical investigators all over the world who have been working earnestly in the field of cell therapy for the past two decades^[1,8]^. Extrapolating the above scandal to cast a pall of suspicion over this entire community of scientists would be not only unfair, but also detrimental to scientific progress, which can only be achieved with continued rigorous investigations, not with avoidance of investigation^[1,2,8]^.

## WHY CLINICAL STUDIES OF CELL THERAPY FOR HF SHOULD CONTINUE

Thus, none of the aforementioned objections is a valid reason to halt clinical testing of cell therapy in HF. On the other hand, there are compelling reasons why basic and clinical, but especially clinical, research on cell therapy should continue.

### 1. Administration of adult cells is safe.

First, and most importantly, cell therapy has proven to be safe. Thousands of patients with various heart ailments have received adult cells, and there have been no reports of serious adverse events caused by the cells in themselves. Adverse events have been reported which were caused by the *delivery* of cells (e.g., cardiac perforation during transendocardial injection), but not by the cell product itself. The experience of 20+ years of research shows therapy with adult cells to be safe. (Parenthetically, this cannot be said of embryonic stem cells^[1,14]^).

### 2. There is Phase II evidence that cell therapy is beneficial.

As mentioned above, several Phase II randomized, double-blind trials have demonstrated the beneficial effects of cell therapy in HF (reviewed in recent papers^[1,2]^). In the setting of ischemic HF, these include TAC-HFT^[35]^, ixCELL-DCM^[30]^, MSC-HF^[31]^, CONCERT-HF^[32]^, and DREAM-HF^[33,34]^. While the design and specific clinical outcome of these trials differ, all of them have shown that a single administration of cells resulted in a measurable clinical improvement 12 months later^[1]^. This is a remarkable outcome. Apart from cardiac resynchronization therapy or implantation of a left ventricular assist device, there is no precedent for a non-device treatment that, given only once, produces long-lasting therapeutic effects detectable 12 months later. Arguably, if the administration of cells could be repeated, the efficacy of cell therapy could be greater. In any case, most researchers would agree that a new treatment that is safe and has proven efficacious in multiple Phase II trials is worthy of investigation in larger Phase III trials^[3]^.

### 3. Heart failure continues to be a major public health problem.

Needless to say, HF is a major health care problem. It is highly prevalent in developed countries, with approximately 6 million patients in the United States alone^[37]^. The prevalence of HF continues to increase as the population ages, and improvements in cardiovascular medicine prolong the lives of patients with heart disease. For patients hospitalized with HF, the 5-year survival is approximately 50%^[37]^. Medical therapy can improve symptoms and survival, but the prognosis of HF remains bleak. Thus, effective treatment of HF is one of the most important unmet needs in contemporary medicine, warranting research into new therapeutic strategies.

## CONCLUSIONS

Herd behavior is the default choice of most human beings, for it is much safer and easier to follow the herd than to think for oneself. It is remarkable (and also frightening) how common herd mentality is even in science - the one domain where fiercely independent thinking should reign supreme. Herd mentality, a.k.a. lack of critical thinking, always promotes error and has had disastrous consequences in the history of science. The current views on cell therapy for HF are one of the latest examples of a misleading herd mentality that has obfuscated the truth.

Many editors, reviewers, funding agencies, biomedical companies, and non-critical thinkers (including, of course, many lay media) seem to agree in unison that cell therapy for HF does not work and should be neither published nor funded. As John Adams said, however, facts are stubborn things. They have a way of sticking around despite widespread misrepresentation. The current tide of hostility toward cell therapy research is not supported by scientific or medical considerations. Contrary to the narratives that are usually encountered in both scientific and lay publications, the results obtained heretofore in clinical trials of cell therapy in HF are encouraging and support a continued investigation. Specifically, therapy with adult cells has proven to be safe, and multiple randomized, placebo-controlled, double-blind studies have shown beneficial effects of a single dose of cells in HF. Although the mechanism of action is unclear, the beneficial effects of cell therapy in these trials were neither incremental nor minor; in fact, they were quite significant^[1,2]^. Given the epidemic proportions of HF and the limitations of current therapies, it would be unreasonable, and possibly unethical, to halt clinical investigation of a promising therapy in this population. It is true that the clinical utility of cell therapy for HF has not been proven with pivotal trials. However, the question as to whether this therapy is effective can be answered only by conducting rigorous, well-designed, Phase III clinical trials. By stifling new research, the ostracism of cell therapy in HF hinders scientific and medical progress. The way forward is not to halt research on the basis of misrepresentations, unwarranted extrapolations, or prejudice, but to subject this therapy to the most rigorous standards of clinical evaluation.
